# Dental macrowear reveals ecological diversity of *Gorilla* spp.

**DOI:** 10.1038/s41598-022-12488-8

**Published:** 2022-06-02

**Authors:** Teagan Harty, Michael A. Berthaume, Eugenio Bortolini, Alistair R. Evans, Jordi Galbany, Franck Guy, Ottmar Kullmer, Vincent Lazzari, Alejandro Romero, Luca Fiorenza

**Affiliations:** 1 Biomedicine Discovery Institute, Department of Anatomy and Developmental Biology, Monash University, Melbourne, VIC Australia; 2 Division Mechanical Engineering and Design, London South Bank University, London, UK; 3 Department of Archaeology and Anthropology, Institución Milá y Fontanals de Investigación en Humanidades, Barcelona, Spain; 4 Culture and Socio-Ecological Dynamics, Department of Humanities, Universitat Pompeu Fabra Ramon Trias Fargas, Barcelona, Spain; 5 School of Biological Sciences, Monash University, Melbourne, VIC Australia; 6 Geosciences, Museums Victoria, Melbourne, VIC Australia; 7 Department of Anthropology, Center for the Advanced Study of Human Paleobiology, The George Washington University, Washington, DC USA; 8 Department of Clinical Psychology and Psychobiology, University of Barcelona, Barcelona, Spain; 9 Laboratory PALEVOPRIM, UMR CNRS 7262, University of Poitiers, Poitiers, France; 10 Department of Paleoanthropology, Senckenberg Research Institute and Natural History Museum Frankfurt, Frankfurt, Germany; 11 Department of Paleobiology and Environment, Institute of Ecology, Evolution, and Diversity, Goethe University, Frankfurt, Germany; 12 Departamento de Biotecnología, Universidad de Alicante, 03690 Alicante, Spain; 13 Instituto Universitario de Investigación en Arqueología y Patrimonio Histórico (INAPH), Universidad de Alicante, 03690 Alicante, Spain

**Keywords:** Biological anthropology, Ecology

## Abstract

Size and shape variation of molar crowns in primates plays an important role in understanding how species adapted to their environment. Gorillas are commonly considered to be folivorous primates because they possess sharp cusped molars which are adapted to process fibrous leafy foods. However, the proportion of fruit in their diet can vary significantly depending on their habitats. While tooth morphology can tell us what a tooth is capable of processing, tooth wear can help us to understand how teeth have been used during mastication. The objective of this study is to explore if differences in diet at the subspecies level can be detected by the analysis of molar macrowear. We analysed a large sample of second lower molars of Grauer’s, mountain and western lowland gorilla by combining the Occlusal Fingerprint Analysis method with other dental measurements. We found that Grauer’s and western lowland gorillas are characterised by a macrowear pattern indicating a larger intake of fruit in their diet, while mountain gorilla’s macrowear is associated with the consumption of more folivorous foods. We also found that the consumption of herbaceous foods is generally associated with an increase in dentine and enamel wear, confirming the results of previous studies.

## Introduction

**Figure 1:**
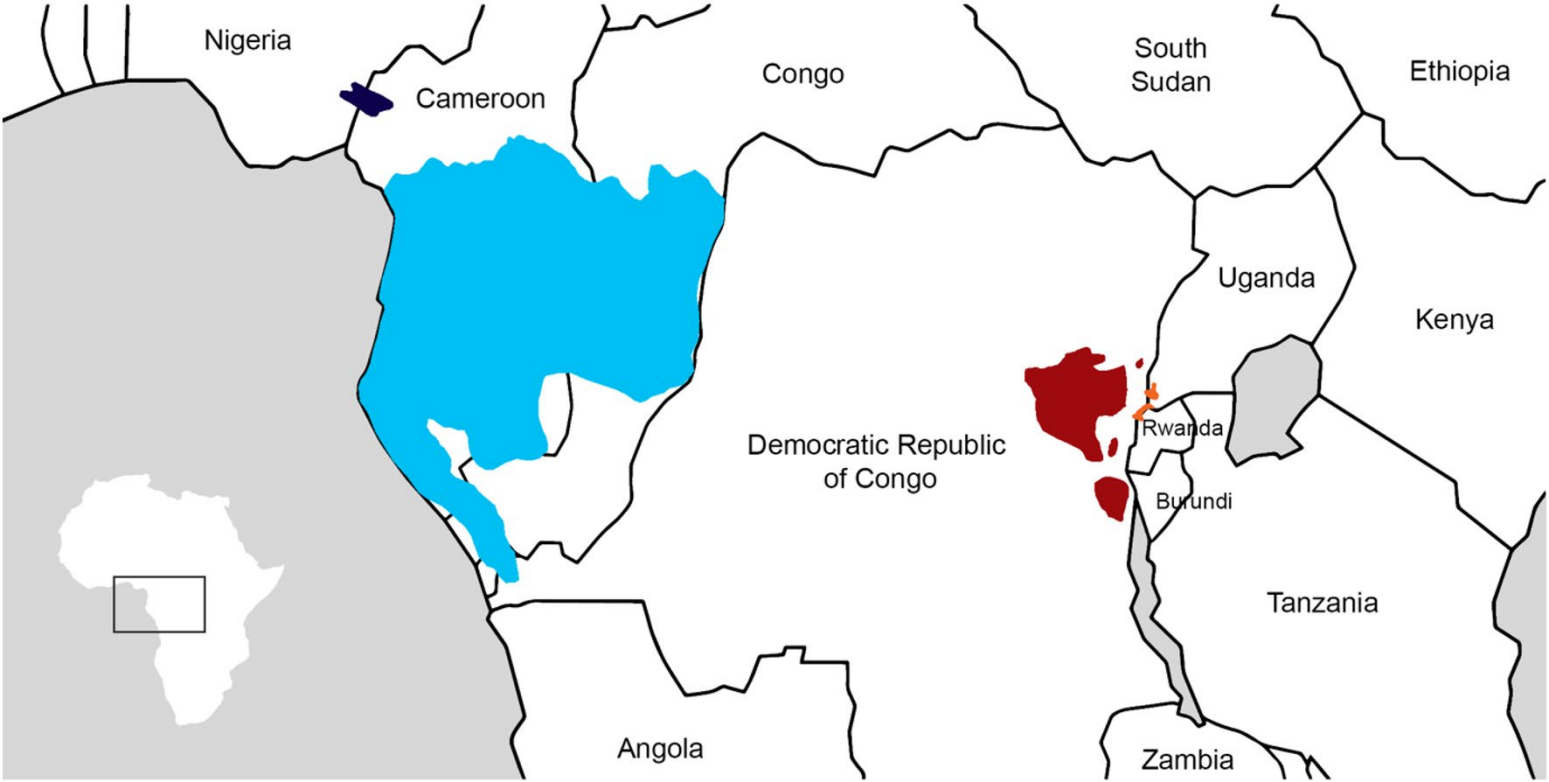
Map of Sub-Saharan Africa showing the distribution of western lowland gorillas (*Gorilla gorilla gorilla*, in light blue), cross river gorillas (*Gorilla gorilla diehli*, in dark blue), Grauer’s gorillas (*Gorilla beringei graueri*, in dark red), and mountain gorillas (*Gorilla beringei beringei, in orange*). Adapted from Xue et al.^5^.

Gorillas have been traditionally described as specialised folivorous primates based on the early behavioural studies from populations of the Virunga Mountains^1,2^. However, additional ecological studies have shown that the most common gorillas, who inhabit western lowland rainforests, eat a considerable variety of foods including large amounts of fruits^3,4^ (Fig. 1).

Gorillas are found in Central and Western Equatorial Africa, and they are separated by the Congo River into western and eastern gorillas^6^. The western gorillas are divided into two subspecies, the western lowland gorillas (*Gorilla gorilla gorilla*; Savage, 1847) and the Cross River gorillas (*Gorilla gorilla diehli*; Matschie, 1904)^7^. The western lowland gorillas are characterised by a coarse and short brown hair that covers most of their body, often red on crown, by flared nostrils, and by a prominent lip above the nasal septum^6,7^. They are geographically and genetically separated from the eastern gorillas, and they are distributed in southern Cameroon, Equatorial Guinea, Gabon, Republic of Congo, northern Angola, and western Democratic Republic of Congo^6,8^. The Cross River gorillas are smaller in size, and differ from western lowland gorillas both in the shape of their cranium and in their genetics^6,7^. They can be found in a small region of highland forests located along the borders of Cameroon and Nigeria. Cross River gorillas are vastly understudied, but data suggests they have diets similar to other western lowland gorillas^9^.

The eastern gorillas consist of two subspecies: mountain gorillas (*Gorilla beringei beringei*; Matschie, 1903), and eastern lowland gorillas, or Grauer’s gorillas (*Gorilla beringei graueri*; Matschie, 1914)^7^. Eastern gorillas are characterised by long black hair, by narrow nostrils, and by the absence of a lip above the nasal septum^6,7^. The mountain gorillas have a large head, wide facial skeleton, and shorter and stockier forelimbs than the western gorillas^6,7^. They live in the high-altitude rainforests of Virunga Volcanoes, along the borders between Rwanda, Democratic Republic of Congo and Uganda, and in the Bwindi Impenetrable Forest between the southwest of Uganda and northwest Rwanda^6^. Grauer’s gorillas have a narrower facial skeleton compared to mountain gorillas and a shorter pelage on the scalp. They can be found in eastern forests of the Democratic Republic of Congo^6,8^.

When rainfall is low and fruit is scarce, gorillas inhabiting lowlands rely on more herbaceous foods, including leaves, stems, pith and barks^10^. These foods fall into the general categories of terrestrial herbaceous vegetation (THV) and aquatic herbaceous vegetation (AHV). Less commonly, other foods, like the woody endocarps of the *Coula edulis* seeds, are incorporated in the diets of some populations of western lowland gorillas, indicating a broader dietary spectrum than previously thought^11^. Both western lowland gorillas and Grauer’s gorillas prefer relatively soft, ripe fruits^10,12^. However, the percentage of THV consumed by western lowland gorillas and Grauer’s gorillas differs, with a larger amount of fibrous food eaten by the eastern populations^13^. On the other hand, mountain gorillas mostly rely on THV foods, which are available year-round, while fruit, especially in the high-altitude forests of the Virunga Mountains, are relatively scarce throughout the year^2,14,15^.

Molar dental studies have found that mountain and Grauer’s gorillas are characterised by molars with duller cusps compared to western lowland gorillas^16^. When duller cusps wear, they create longer compensatory crests, increasing the contact areas between the sharp blade-like crests on the molars and the food particles. This provides a functional advantage compared to molars with sharper cusps, as it increases the cutting surfaces for processing fibrous foods^17^. Another study utilised the Dirichlet normal energy (DNE; a dental topographic method) to analyse the diet of seven groups of great apes, finding a positive correlation between DNE and fibre content in apes among sympatric pairs^18^. DNE is a measure of surface curvature, meaning teeth with higher DNE values are curvier, or sharper, potentially making it easier to break down foods with a high fibre content^19^. The presence of character displacement in DNE suggests the relationship between tooth curvature and fibre content is affected not only by diet, but also other parameters, such as indirect interspecific competition over food resources.

Moreover, a study based on buccal microwear signals found geographic differences between various populations of Grauer’s gorillas which were attributed to ecological conditions^20^. Dental microwear texture analyses have compared western lowland gorillas with mountain gorillas without finding any clear differences between the two groups^21^. This is probably due to dietary overlap between mountain and lowland gorillas, which may be large enough that microwear differences are not observable. In addition to this, tooth microwear signatures can change very rapidly, yielding information only about an individual’s diet in the weeks or even days before its death^22^. In turn, a recent study in chimpanzees revealed that dental microwear texture can provide information about short-term dietary changes, such as seasonality, but are less effective in providing long-term dietary signals^23^. Elgart^24^ correlated the toughness of food with the amount of dental wear in *Gorilla*, finding some inconclusive results in relation to their diet, potentially due to complications in gathering dietary mechanical property data^25^. Galbany and colleagues^26^ measured the percentage of dentine exposure (PDE) in permanent molars of mountain gorillas from the Volcanoes National Park (Rwanda), finding a significant relationship between the degree of tooth wear with the consumption of plant roots at the individual level.

However, we still do not comprehensively understand if ecological constraints and dietary differences observed in gorilla subspecies are reflected in tooth wear. The aim of this study is to explore if differences in diet in gorillas can be detected at the subspecies level by using molar macrowear analyses in combination with various dental measurements.

Dental macrowear, as opposed to dental microwear, is a cumulative process, which occurs during the individual’s lifetime and thus reflects long-term dietary and environmental history^27,28^. Here we examine the macrowear patterns of second lower molars (M2s) in a large sample of western lowland gorilla, Grauer’s gorillas and mountain gorillas by combining the Occlusal Fingerprint Analysis method (OFA)^29^, a well-established digital approach tracking changes in dental function by examining occlusal wear facets (planar areas with well-defined edged boundaries produced by the attritional and abrasional contact between upper and lower teeth), with other dental measurements, including the occlusal relief index (OR), PDE and percentage of enamel wear (PEW).

OFA has been used to reconstruct the diet and chewing behaviour of past human populations and in extinct and living non-human primates^28,30–35^. The proportions of crushing, grinding and shearing wear can be used to better discriminate between population with different diets, while tooth wear inclination provides information about the abrasiveness of the diet^31,34,36^. For example, Stuhlträger and colleagues^35^ found that the inclination and size of wear facets in two chimpanzee populations were positively correlated with the abrasives in their diet, while a study of molar wear angles of phase II facets revealed significant differences among great ape species, with higher degree of inclination correlated with the consumption of tough and pliant foods such as leaves^28^. Moreover, OFA has also been used to identify unusual types of wear, revealing an unexpected case of bruxism (grinding the teeth and clenching the jaw) in a wild adult male gorilla characterised by the presence of a supernumerary maxillary premolar^33^.

OR has been widely used to reconstruct primate diets, and it is based on the assumption that folivorous species should be characterised by a high relief because it increases the efficacy in processing fibrous and tough foods such as leaves^37^. PDE is a method that can be employed to obtain information about the interaction between dental tissue with various ecological factors, including diet^26,38^, while PEW is a new measurement that aims to quantify the volume of wear in a different way.

Previous molar macrowear studies in Pleistocene and Holocene humans, colobus monkeys, and ungulates, have shown that the more fibrous the diet, the greater is the proportion of buccal phase I facets^27,30^, while the consumption of softer foods in primates, such as fruit, is generally associated with the presence of larger lingual phase I facets^27^. Thus, we expect to find larger buccal shearing wear in the folivorous mountain gorillas, and larger lingual shearing wear in the more frugivorous Grauer’s and western lowland gorillas. We also expect to find a more complex occlusal morphology (higher OR values) with sharper cusps (higher wear inclinations) in mountain gorillas enhancing comminution of fibrous foods such as leaves and stems. PDE in primates seems to be proportionally correlated with the amount of grit and dust inadvertently ingested with foods^26,38^. Because mountain gorillas consume a larger amount of THV foods, which are covered by soil and dust from the sediments, they should exhibit higher PDE values compared to Grauer’s and western lowland gorillas.

We will also test, using PEW, if western lowland gorillas are characterised by the highest degree of wear as suggested by previous studies^24^. Finally, we also explore if there are any differences in molar macrowear between males and females of western lowland gorillas. Gorillas are characterised by a marked sexual dimorphism, with adult males often weighing twice as much as females^39^. This marked body size difference is reflected also in the nutritional requirements, with males spending more time in feeding than females^40^. Because prolonged chewing times have an effect on tooth wear, we expect to find a greater amount of wear in males than in females.

## Results

**Figure 2:**
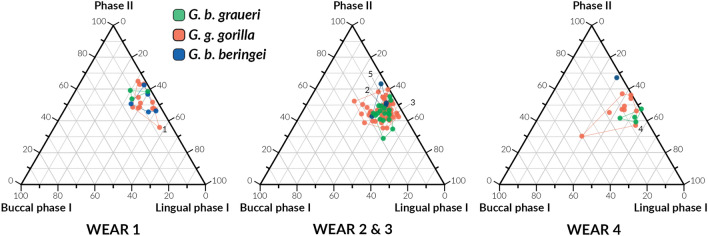
Ternary diagrams showing the proportions (in %) of relative wear areas of buccal phase I facets, lingual phase I facets, and phase II facets, which are positioned in an equilateral triangle. Each base of the triangle represents a ratio of 0% while the vertices correspond to a percentage of 100%. Tip crush areas were included in phase II facets. *Gorilla beringei graueri* in green, *Gorilla gorilla gorilla* in orange, and *Gorilla beringei beringei* in blue. Specimens 1 = AMNH 115609, 2 = AMNH 200506, 3 = SMF 59159, 4 = ZMB 47526, and 5 = BM 64.12.1.5.

Overall, gorillas are characterised by small buccal phase I facets, and larger phase II and lingual phase I facets (Table 1). During the earlier wear stages, the macrowear patterns of the three gorilla groups look similar, with differences emerging only at the later wear stages (Fig. 2).

**Table 1:** Summary statistics (median and SD) of relative wear facet areas divided by wear stages.

	N	Buccal P I		Lingual P I		PII	
		Median	SD	Median	SD	Median	SD
**Wear 1**							
*G. b. beringei*	5	0.04	0.05	0.39	0.07	0.51	0.08
*G. b. graueri*	3	0.11	0.06	0.33	0.05	0.49	0.03
*G. g. gorilla*	13	0.05	0.04	0.38	0.08	0.48	0.08
**Wear 2 and 3**							
*G. b. beringei*	4	0.06	0.07	0.40	0.04	0.51	0.09
*G. b. graueri*	18	0.11	0.04	0.45	0.04	0.45	0.06
*G. g. gorilla*	54	0.09	0.06	0.44	0.06	0.47	0.05
**Wear 4**							
*G. b. beringei*	1	0.03	–	0.30	–	0.67	–
*G. b. graueri*	4	0.07	0.06	0.53	0.05	0.42	0.03
*G. g. gorilla*	10	0.08	0.13	0.44	0.06	0.48	0.08

The macrowear pattern of mountain gorillas is dominated by large phase II facets (between 51 and 67%), followed by lingual (between 30 and 40%) and buccal phase I facets (between 3 and 6%). The macrowear pattern of Grauer’s gorillas is rather homogenous compared to the other two groups and is characterised by large lingual phase I facets (between 33 and 53%), followed by phase II facets (between 42 and 49%), and by buccal phase facets (between 7 and 11%). Finally, western lowland gorillas show the most variable molar macrowear pattern, with large lingual phase I facets (between 38 and 44%) and phase II facets (between 47 and 48%), and small buccal phase I facets (between 5 and 9%). This was confirmed by the Levene’s test of equality of variance among the three gorilla groups (Table S1).

No statistically significant differences were found when comparing molars with wear stage 1 (Table S2 and S3).

Statistically significant differences were found when comparing lingual phase I facets of mountain gorillas with those of Grauer’s gorillas (*p* = 0.040; Wear stage 2 and 3). No differences have been found between molars with wear stage 4. However, the only mountain gorilla with wear 4 included in this analysis looks very different compared to Grauer’s and western lowland gorillas (Fig. 2).

Gorilla molars are generally characterised by steep facets, with average angles of phase I and phase facets comprised between 26 and 47 degrees (Table 2). Tip crush areas are relatively flat, with values comprised between 8 and 16 degrees. Phase II facets show much steeper angles if compared to phase I facets, especially in slightly worn molars. The opposite situation is found in more heavily worn molars with phase I facets characterised by higher values if compared to the planes of phase II facets. The mountain gorillas generally display the steepest wear facets, while western lowland and Grauer’s gorillas tend to have more variable inclinations depending on the wear stage considered (Table S4 and S5).

**Table 2:** Summary statistics (median and SD) of wear facet inclinations divided by wear stages.

	N	Buccal P I		Lingual PI		Phase II		Tip crush	
		Median	SD	Median	SD	Median	SD	Median	SD
**Wear 1**									
*G. b. beringei*	5	32.76	6.54	38.03	4.56	46.5	6.01	16.42	7.32
*G. b. graueri*	3	32.00	7.84	36.66	3.90	41.94	1.88	0.00	12.48
*G. g. gorilla*	13	29.43	6.02	40.55	4.40	44.75	4.70	0.00	9.54
**Wear 2**									
*G. b. beringei*	2	24.23	0.35	31.49	1.44	41.98	1.040	11.87	16.78
*G. b. graueri*	9	35.01	2.55	36.74	5.75	38.97	5.08	11.36	8.17
*G. g. gorilla*	27	29.40	5.17	38.62	5.40	37.4	4.63	15.94	7.37
**Wear 3**									
*G. b. beringei*	2	39.58	10.63	37.00	14.26	32.83	2.46	10.06	3.49
*G. b. graueri*	9	31.90	8.96	35.68	2.56	34.83	4.62	12.12	1.73
*G. g. gorilla*	27	32.73	7.44	37.30	3.72	32.23	5.53	13.13	5.99
**Wear 4**									
*G. b. beringei*	1	36.81	–	30.64	–	33.40	–	7.75	–
*G. b. graueri*	4	28.40	15.49	37.57	2.64	28.70	1.59	11.46	5.50
*G. g. gorilla*	10	26.64	8.12	34.71	3.42	29.28	7.73	14.49	4.67

We could not statistically test if mountain gorillas facet inclinations differ from those of Grauer’s and western lowland gorillas, because we could not group together molars with wear stage 2 with molars with wear stage 3. We could only compare the wear facet angles of molars with wear stage 1, which did not produce any statistically significant difference (Table S6 and S7). The only statistically significant difference has been found between the buccal phase I facets of Grauer’s and western lowland gorillas (*p* = 0.016; Wear 2).

Mountain gorillas display lower OR values compared to those of western lowland gorillas (Table 3).

**Table 3:** Summary statistics (median and SD) of occlusal relief index (OR), percentage of dentine exposure (PDE) and percentage enamel wear (PEW).

Groups	N	OR		PDE		PEW	
		Median	SD	Median	SD	Median	SD
*G. b. beringei*	10	1.895	0.133	0.245	3.156	20.215	8.540
*G. b. graueri*	25	1.990	0.190	1.660	3.550	24.290	6.600
*G. g. gorilla*	77	2.040	0.268	0.760	3.400	26.830	7.760

Overall, western lowland gorillas are also characterised by the highest degree of variation in OR, while the least variable are the mountain gorillas. Grauer’s gorillas display intermediate OR values. No statistically significant differences have been found in OR values among the three gorilla groups examined in this study (Table S8).

In terms of PDE, Grauer’s gorilla show the highest values, followed by western lowland gorillas and mountain gorillas (Table 3). No statistically significant differences have been found when comparing PDE values between the gorillas examined in this study (Table S8).

Western lowland gorillas are generally characterised by the highest PEW values, followed by Grauer’s and mountain gorillas (Table 3). We found statistically significant differences in PEW values among the three gorilla groups (*p* = 0.016; Table S8). Finally, no statistically significant differences have been found between males and females of western lowland gorillas for all variables considered in this study (Tables S10–S13).

## Discussion

We have found significant differences in molar macrowear patterns between mountain gorillas and lowland gorillas, confirming our initial hypothesis. Grauer’s and western lowland gorilla molar macrowear patterns are dominated by lingual phase I facets, which is probably associated with a larger intake of fruit in their diet. Previous studies of molar macrowear on various ungulate mammals and of colobus monkeys suggested that lingual phase I facets are linked to the consumption of softer food items such as fruit^27^. However, we found that Grauer’s gorillas display the largest lingual phase I facets, especially at the most advanced wear stages. We would have expected to find the largest lingual phase I facets in western lowland gorillas, considering their more frugivorous diet^41^. We observe this trend only when examining slight worn molars, while at intermediate levels of wear the proportion of lingual phase I facets in Grauer’s and western lowland gorillas is similar. Larger lingual phase I facets are possibly correlated with greater transverse mandibular movements, which may also indicate the consumption of foods other than fruit such as roots, seeds and gums^27,30^. Mountain gorilla molars are characterised by the largest phase II facets which seem to be associated with a greater consumption of THV foods. Contrary to previous findings, where higher degrees of folivory was linked to larger buccal phase I facets^27^, here we found that the consumption of herbaceous food is associated with an increase of the proportion of phase II facets and tip crush areas. Buccal phase I facets are very small in all gorillas examined in this study at any given wear stage. This seems to be very common in all great apes, which would indicate a distinctive masticatory behaviour compared to early and late hominins^42^. The functional significance of phase II facets is still highly debated^43^. Phase II facets are used during both phases of mastication, including crushing (phase I) and grinding (phase II)^44^. Grinding is a combination of forces that act parallel and perpendicular to the contact planes producing a more horizontal shear force^45^. Consequently, the consumption of THV foods, which requires prolonged exposure to repetitive mastication^46^, probably promote the formation of large phase II facets.

As expected, the western lowland gorillas show the highest degree of macrowear variation, suggesting a more flexible diet if compared to those of eastern gorillas. Western lowland gorillas occupy a large geographical area, ranging between the southern part of Cameroon to North Angola^8^. They also inhabit a variety of lowland forest types, including open and closed-canopy forests, and vast swamps in northern Congo^6^. Western lowland gorillas eat a wide variety of fruit, preferring ripe, succulent, sweet fruits that are low in proteins and fat^4^. However, fruit productivity is highly seasonal, and therefore the preferred food of western lowland gorillas is not always available. During these periods of fruit scarcity, western lowland gorillas consume larger quantity of THV, including pith, shoots, leaves and barks^41^. Moreover, some populations of western lowland gorillas regularly consume AHV, which are high in essential vitamins and minerals^4,8^, while evidence of seasonal hard-object feeding has been documented in one population of western lowland gorillas from Loango National Park, Gabon^11^.

Grauer’s gorillas and the mountain gorillas show a less variable macrowear pattern suggesting the intake of a less diverse diet if compared to western lowland gorillas. Mountain gorillas occupy high altitude forests, which are characterised by a significantly lower diversity of plants and fewer fruits^46^. Grauer’s gorilla’s diet is more variable than those of mountain gorillas. At higher altitude the diet mostly consists of THV foods, like mountain gorillas, while the populations from lower elevations incorporate more fruit in their diets^3,6^.

Mountain gorillas are generally characterised by steeper phase II facets, probably increasing the shearing capabilities, which is functionally advantageous in processing tough and pliant foods such as leaves^28^. This is very different from the wear patterns observed in late *Homo*, where phase II facets are significantly flatter than buccal and lingual phase I facets^31^. Significant differences in inclination of buccal phase I facets between Grauer’s and western lowland gorillas are probably linked to the consumption of THV foods. Although the buccal phase I facets play a little role in gorilla chewing behaviour, their steeper planes in Grauer’s gorillas increase their shearing capacity, more suitable for processing herbaceous vegetation. We observe the same pattern in mountain gorillas, but because of the small sample size we could not test if there were any statistically significant differences with western lowland gorillas.

While the percentage of dentine exposure is negligible in slightly worn molars, we noticed larger PDE values in more advanced wear stages in the mountain and Grauer’s gorillas. This could be correlated with the increased amount of THV foods included in their diet. Feeding time in primates increases as toughness increases^41^. A previous study has shown that PDE in mountain gorillas from the Virunga National Park was positively correlated with the time spent consuming plant roots^26^. This was also probably correlated with the amount of exogenous material accidentally introduced in their diet, which is more likely to cause increased tooth wear^42^. It has been observed that mountain gorillas pull the plants out from the soil to access the roots without properly eliminating all the gritty particles that cover the plants^26^. We have also observed that the molars of western lowland gorillas are generally characterised by a higher occlusal relief index compared to those of mountain and Grauer’s gorillas, partially confirming the results of Berthaume’s study^16^ who found that western lowland gorillas were characterised by sharper cusps compared to mountain and Grauer’s gorillas.

Mountain gorillas are characterised by the least amount of wear (PEW) compared to western lowland and Grauer’s gorillas, confirming the results of previous studies^24,26^. This would indicate that mountain gorilla molars maintain a good chewing efficiency throughout the life of an individual despite the mechanically demanding diet^24^. Similarly, the high PEW values of western lowland gorillas are in agreement with Elgart’s study^24^, where it was found that the western populations showed the highest quantities of wear.

Finally, dental topographic and macrowear analyses of western lowland gorilla molars did not reveal any difference between males and females, rejecting thus our initial hypothesis. Previous studies support these results as it was found that there were no differences in occlusal relief and in the amount of wear between males and females of western lowland gorillas^24,37^. Previous studies have also identified that western lowland gorilla males in Bai Hoköu, Central African Republic, spend less time feeding and more time resting compared to females and immatures in their group^41^.

It is important to consider some limitations in our current study. First of all, the sample size of mountain gorillas is rather small, and therefore our interpretations need to be taken with caution. Unfortunately, most of the museum collections we examined included only a small number of mountain gorillas. Their teeth were often heavily worn and chipped, preventing thus their inclusion for the occlusal fingerprint analysis.

Statistically testing the variability across samples of unequal sizes can be problematic. The largest variation found in the western lowland gorillas could be simply driven by the larger sample size, and may not reflect the ecology of this taxon. To overcome this limitation, we have performed additional statistical tests, such as the Permutational Multivariate Analysis of Variance, which confirmed that western lowland gorillas are characterised by the most variable macrowear pattern. We also verified that the variance found in the groups analysed in this study is homogenous, which should have partially minimised this problem.

Because our sample mostly consists of museum specimens, the age of the individual was often unknown, and the geography was frequently uncertain. This additional information could have been very useful with the interpretation of the results. For example, a recent study of dental wear patterns in chimpanzee populations from precise geographic locations revealed previous information for the ecological interpretations of their results^23^. In relation to this, more detailed information on how the sample has been acquired could have helped to better interpret the results. Because most of the sample analysed in this study has been wild-shot, and therefore did not live a full life, we should expect a less amount of wear compared to those individuals who have died of natural causes. Moreover, a couple of specimens, caught in the wild, actually died in zoos. A closer look at their macrowear patterns revealed some differences, especially for the specimens SMF 59159 and ZMB 47526, who died at the zoos of Duisburg and Berlin respectively^47^ (Fig. 2). These specimens are characterised by a slightly larger percentage of lingual phase I facets, which may reflect a higher intake of fruit in their diet. The diet of great apes living in captivity has been traditionally based on cultivated fruits and vegetables, pellet concentrate and browse^48^. It would be interesting to examine if macrowear patterns of apes grown in captivity differ from those caught in the wild considering they consume different diets. In addition, future studies could include a larger sample of mountain gorillas, and could also compare the analysis of permanent and deciduous dentition to examine if there are any dietary difference between adult and immature individuals.

## Materials and methods

### Ethical statement

No live animals were handled in this study.

The sample examined consists of 112 gorilla specimens, including 10 *Gorilla beringei beringei*, 25 *Gorilla beringei graueri*, and 77 *Gorilla gorilla gorilla*. We selected only fully erupted M2s (either left or right, but no antimeres) because they provide a good general overview of the development of masticatory function in primates^49^. We included only molars with a slight to moderate degree of wear because in heavily worn teeth occlusal facets tend to coalesce often preventing a clear identification^30^. We qualitatively evaluated the level of wear based on the amount of dentine exposure and cusp removal (wear stages 1–4)^50^. When available we included from museum records^47^ information about age (subadult and adult), sex, locality and acquisition (electronic supplementary material, Table S14). The sample mostly consists of specimens captured in the wild (wild-shot). Unfortunately, it was difficult to obtain additional information about the way the sample has been collected. We did not include any information about their acquisition for the specimens that either did not have any acquisition date, or that have been acquired after 1950, because it was difficult to establish if the sample has been collected opportunistically or hunted by local people for bushmeat. There are only a handful specimens that died in zoos, but that have been collected in the wild. Some of these specimens, such as BM 64.12.1.5, may have not survived the journey when they arrived at their destination^47^.

### Occlusal fingerprint analysis (OFA)

Three-dimensional (3D) digital models of teeth were post-processed using Polyworks V12 (InnovMetric Software), a 3D metrology software. OFA consists of four consecutive steps: (1) model orientation, (2) facet identification, (3) facet area, and (4) facet inclination^29^. The polygonal models are oriented using a reference plane that is created along the cervical line of the tooth through the least square best-fit method. Successively, the reference plane is rotated to the *xy* plane obtained from the original coordinate system.

**Figure 3:**
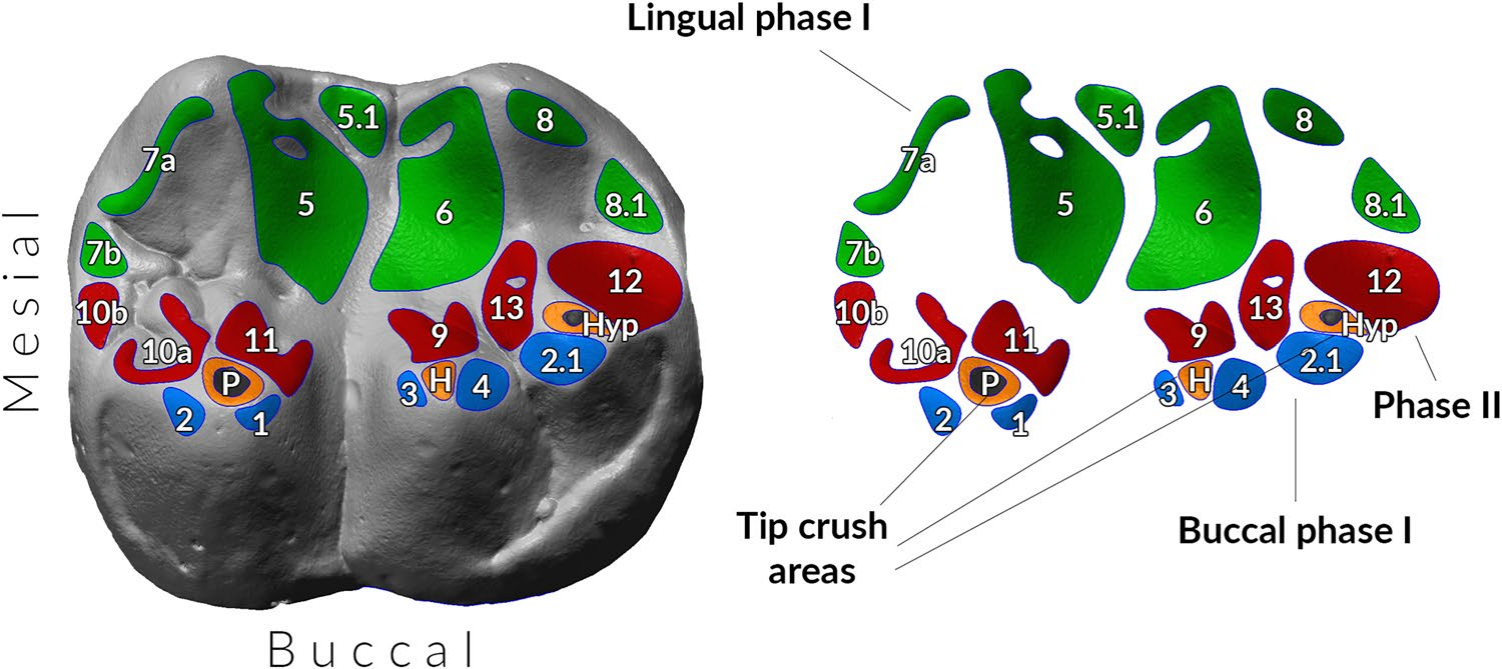
Second right lower molar of a western lowland gorilla adult male (90,194) showing the macrowear pattern. Facets are numbered according to the Maier and Schneck’s labelling system^51^. Buccal phase I (facets 1, 2, 2.1, 3 and 4; in blue), lingual phase I (facets 5, 5.1, 6, 7, 8 and 8.1; in green) and phase II (facets 9, 10, 11, 12 and 13; in red). We also identified tip crush areas (in orange) and dentine exposures (in dark grey). P = protoconid, H = hypoconid, Hyp = hypoconulid.

The identification of wear facets follows the numbering system originally created by Maier and Schneck^51^ and later modified by Kullmer and colleagues^29^. Facets 1, 1.1, 2, 2.1, 3 and 4 develop along the buccal slopes of protoconid, hypoconid and hypoconulid, while facets 5, 6, 7 and 8 form along the buccal slopes of the metaconid and entoconid (Fig. 3). These facets are created during the initial phase of the rhythmic chewing cycle (phase I), and they can be further divided into buccal (facets 1–4) and lingual (facets 5–8) facets^44^. Facets 9, 10, 11, 12 and 13 are formed during the second phase of the rhythmic chewing cycle (phase II), when the mandibular molars move out of occlusion. These facets develop along the lingual slopes of protoconid, hypoconid and hypoconulid.

During the rhythmic chewing cycle, the food bolus is initially processed by shearing, which is generated by forces parallel to the contact plane (phase I), followed by crushing between basins and cusps of molars, where the occlusal force is perpendicular to the contact plane^44^. After the molars reach centric occlusion (phase II), the food bolus is processed by grinding, which is the resulting action of the combination of perpendicular and parallel forces to the contact plane.

We also identify tip crush areas, which are generally formed during puncture-crushing, when food is initially pulped by a series of masticatory cycle where tooth-to-tooth contacts do not occur^27^. Finally, we identify three additional facets in gorilla molars: facet 5.1, facet 8.1 and facet 10.1. These facets are created by the presence of additional dental traits, which are less common in *Homo*. More specifically, facets 5.1 forms in mandibular molars around the area of cusp 7 (C7), and it occludes with maxillary molars in proximity of the lingual cingulum^32^. Facet 8.1 forms around cusp 6 (C6) and it occludes with the lingual cingulum of the maxillary molars in correspondence of the mesiolingual aspect of the protocone^33^. Facet 10.1 has been previously described in Neanderthal molars, and it is created by the contact between the distolingual slope of the metaconule of the maxillary molar with the mesiolingual slope of the protoconid^52^. Facets 5.1 and 8.1 are grouped with lingual phase I facets, while facet 10.1 is considered a phase II facet.

Once the facets have been identified, we automatically calculate the surface area using the *area* function available in Polyworks V12 (InnovMetric Software). To facilitate the analysis of the occlusal contact areas we grouped phase II facets together with tip crush areas, and divided the phase I facets into buccal and lingual facets^30^. However, tip crush areas and phase II facets have been kept separated for the analysis of facet inclination, considering they are characterised by significantly different angles^31^. Because larger teeth generally develop larger facets than smaller teeth, we eliminate the size factor by using relative areas only. Relative areas are calculated by dividing the absolute area of a facet with the total wear area (TWA), which is the sum of absolute area of all facets^30^. The facet inclination is calculated by measuring the angle between the reference, or cervical plane, with the facet plane, which is created by selecting all triangles within its perimeter and by applying the best-fit plane function of Polyworks V12 (InnovMetric Software) (electronic supplementary material, figure S1).

### Occlusal relief index (OR)

The occlusal relief index (OR) was calculated by dividing the 3D area by the two-dimensional (2D) area of the occlusal surface^53^. The occlusal plane, parallel to the cervical plane, was translated along the *y* axis until it reached the deepest point of the occlusal surface, known as the central fossa. Next, the digital model was sliced, with respect to the occlusal plane, and the 2D area was calculated. The 3D area was calculated by selecting all triangles of the polygonal models above the occlusal plane (electronic supplementary material, figure S2).

### Percentage of dentine exposure (PDE) and enamel wear (PEW)

To calculate the percent of dentine exposure (PDE) is obtained by dividing the sum of total dentinal areas for each tooth by the 3D occlusal area and then multiplied by 100^26^. The percentage of enamel wear (PEW) is obtained by dividing the total wear area (TWA) with the 3D occlusal area and then multiplied by 100.

### Statistical analysis

We employed summary statistical analyses (median and standard deviation, SD) for each variable considered in this study. Because we further divided our sample according to wear stages 1 to 4^50^ the comparative groups became relatively small. As the presence of a small sample size prevents the assumption of a normal distribution, we used the nonparametric Mann–Whitney pairwise test, to test whether two univariate samples are taken from populations with equal medians^54^. For the analysis of facet areas, we grouped together molars characterised by wear stage 2 and wear stage 3 in order to maximize the sample size. We then tested if we could combine these two groups by comparing molars wear stage 2 with molars wear stage 3 of western lowland gorillas without finding any statistically significant result (Table S15). However, for the inclination we kept the molars separated by their wear stages, because the level of wear strongly influences the values of these variables^31^. OR, PDE and PWE were not separated into different wear stage groups.

Difference across groups in the distribution of individual variables expressed as percentages was calculated after running arcsine transformation. This method was preferred over logit transformation because of the presence of zeros that would have otherwise generated negative infinite values. Because of the heavily unbalanced sample sizes, and of the potential violation of parametric assumptions, we only relied on non-parametric tests. Homoscedasticity across groups was ascertained through the Levene’s Test of equality of variances using the robust Brown–Forsythe Levene-type procedure (i.e. the median)^55^ and the combination of O'Brien's correction factor and Hines–Hines structural zero removal^56^ contained in the package lawstat in R version 4.1^57,58^. After having controlled for homogeneity of variances due to unequal sample sizes across the sample, a Kruskal–Wallis test was used to test for the presence of significant differences in the distribution of wear areas within each wear stage and between the gorilla groups. Significant cases were further explored via a Dunn test with Benjamini-Hochberg (BH) correction to quantify pairwise relationships. The same procedure was used to test for differences in the distribution of OR, PDE, and PEW between geographic groups.

All variables expressing wear facet inclinations were used to calculate a pairwise Euclidean distance. Heteroscedasticity within the sample was then calculated on the distance matrix using the analysis of multivariate homogeneity of group dispersion (function betadisper in the package vegan in R, 999 permutations)^59^, Tukey’s Honest Significant Differences test, and permutation test for homogeneity of multivariate dispersion (function permutest in package vegan, *n* = 999). Difference between the gorilla groups within each wear stage class was then calculated through a Permutational Multivariate Analysis of Variance (Permanova; 999 permutations) using the function adonis in the package vegan in R.

Differences between male and female individuals of the western lowland gorilla were tested via two-tailed Mann–Whitney tests for independent sample design, while differences in wear facet area of western lowland gorillas across wear stage classes was ascertained through a pairwise Permanova using the package pairwise Adonis^60^. The summary statistical analyses, ternary diagram and Man-Whitney test were performed using the software PAST v.3.22 (PAlaeontological Statistics)^61^, while for all other statistical analyses we used the R software^58^.

## Supplementary Information

Supplementary Information.
