# Supplementary material for: Dental macrowear reveals ecological diversity of Gorilla spp
Source: Sci Rep. 2022 Jun 2;12:9203. doi: 10.1038/s41598-022-12488-8 (PMC9163330; doi:10.1038/s41598-022-12488-8)
Supplement: Supplementary file 1 — Supplementary Information. [file 41598_2022_12488_MOESM1_ESM.docx]

**Supplementary Information**

**
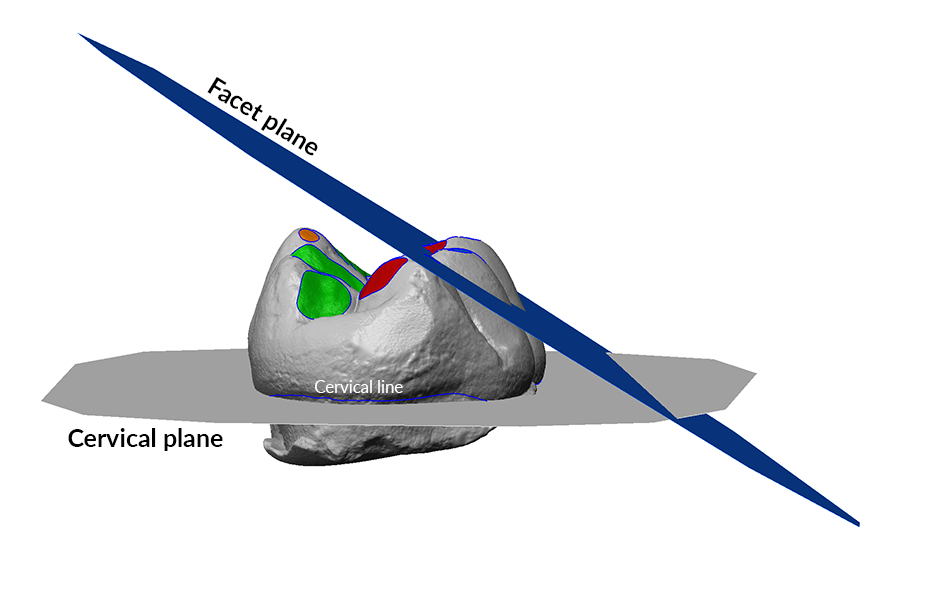
**

**Figure S1.** Three-dimensional (3D) polygonal model of a lower second mandibular molar of an adult western lowland gorilla (SMF 45713) showing the wear facet inclination (measured as the angle between the facet plane and the cervical plane). Buccal: right.

**
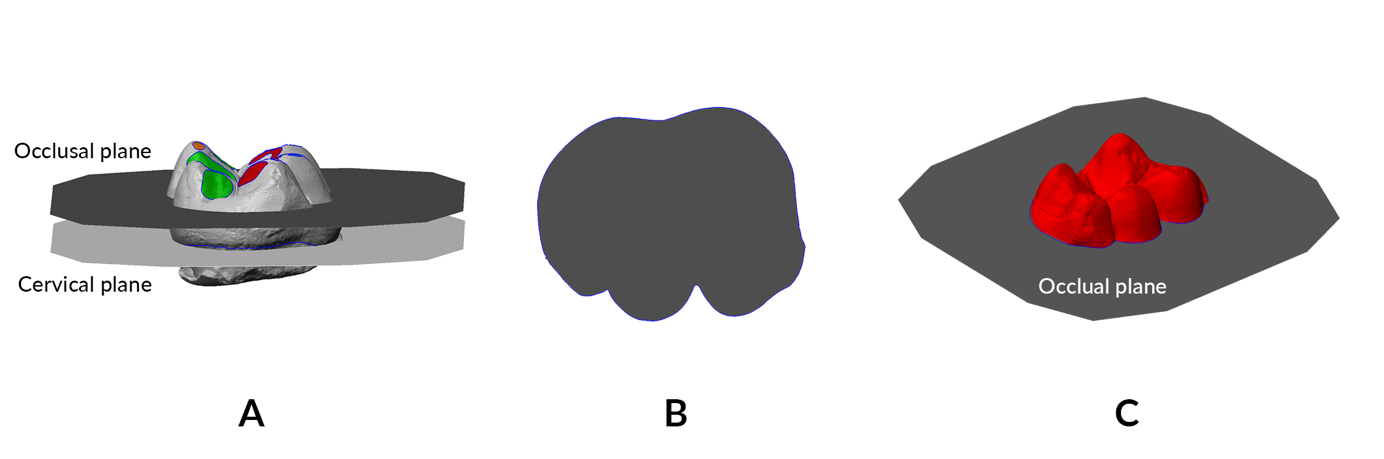
**

**Figure S2.** Occlusal Relief Index (ORI). Translation of the cervical plane along the y-axis reaching the deepest point of the occlusal surface (A), two-dimensional (2D) occlusal area (B), and 3D occlusal area (C).

**Table S1.** Levene’s test of equality of variances between mountain gorillas (*G. b. b.*), eastern lowland gorillas (*G. b. g.*) and western lowland gorillas (*G. g. g*.) for each wear class.

|  | **Buccal** | | **Lingual** | | **PhaseII** | |
| --- | --- | --- | --- | --- | --- | --- |
| **Wear** | **Test statistic** | **p-value** | **Test statistic** | **p-value** | **Test statistic** | **p-value** |
| Wear 1 | 0.28 | 0.76 | 0.26 | 0.77 | 0.47 | 0.63 |
| Wear 2+3 | 0.72 | 0.49 | 0.38 | 0.68 | 0.6 | 0.55 |
| Wear 4 | 0.006 | 0.94 | 0.28 | 0.61 | 1.38 | 0.27 |

**Table S2.** Kruskal-Wallis test of differences in relative wear facet areas between mountain gorillas (*G. b. b.*), eastern lowland gorillas (*G. b. g.*) and western lowland gorillas (*G. g. g*.) for each wear class.

|  | **Buccal** | | | **Lingual** | | | **Phase II** | | | |
| --- | --- | --- | --- | --- | --- | --- | --- | --- | --- | --- |
| **Wear** | **KW Χ^2^** | **df** | **p-value** | **KW Χ^2^** | **df** | **p-value** | **KW Χ^2^** | **df** | **p-value** |  |
| Wear 1 | 0.4 | 2 | 0.81 | 2.22 | 2 | 0.33 | 1.73 | 2 | 0.42 |  |
| Wear 2+3 | 2.09 | 2 | 0.35 | 4.27 | 2 | 0.12 | 3.04 | 2 | 0.22 |  |
| Wear 4 | 0.94 | 2 | 0.62 | 5.9 | 2 | 0.051 | 4.21 | 2 | 0.12 |  |

**Table S3.** Between-group comparisons (Mann-Whitney test) of relative wear facet areas. Mountain gorillas (*G. b. b.*), eastern lowland gorillas (*G. b. g.*) and western lowland gorillas (*G. g. g*.). Significant differences at *p*<0.05 are highlighted in bold.

| **Wear 1** |  |  |  | **Wear 2&3** |  |  |  | **Wear 4** |  |  |  |
| --- | --- | --- | --- | --- | --- | --- | --- | --- | --- | --- | --- |
| **Buccal** | ***G. b. b.*** | ***G. b. g.*** | ***G. g. g.*** | **Buccal** | ***G. b. b.*** | ***G. b. g.*** | ***G. g. g.*** | **Buccal** | ***G. b. b.*** | ***G. b. g.*** | ***G. g. g.*** |
| *G. b. b.* | - |  |  | *G. b. b.* | - |  |  | *G. b. b.* | - |  |  |
| *G. b. g.* | 0.881 | - |  | *G. b. g.* | 0.267 | - |  | *G. b. g.* | nd* | - |  |
| *G. g. g.* | 0.618 | 0.684 | - | *G. g. g.* | 0.295 | 0.348 | - | *G. g. g.* | nd | 0.620 | - |
| **Lingual** | ***G. b. b.*** | ***G. b. g.*** | ***G. g. g.*** | **Lingual** | ***G. b. b.*** | ***G. b. g.*** | ***G. g. g.*** | **Lingual** | ***G. b. b.*** | ***G. b. g.*** | ***G. g. g.*** |
| *G. b. b.* | - |  |  | *G. b. b.* | - |  |  | *G. b. b.* | - |  |  |
| *G. b. g.* | 0.177 | - |  | *G. b. g.* | **0.040** | - |  | *G. b. g.* | nd | - |  |
| *G. g. g.* | 0.844 | 0.225 | - | *G. g. g.* | 0.061 | 0.630 | - | *G. g. g.* | nd | 0.079 | - |
| **PII** | ***G. b. b.*** | ***G. b. g.*** | ***G. g. g.*** | **PII** | ***G. b. b.*** | ***G. b. g.*** | ***G. g. g.*** | **PII** | ***G. b. b.*** | ***G. b. g.*** | ***G. g. g.*** |
| *G. b. b.* | - |  |  | *G. b. b.* | - |  |  | *G. b. b.* | - |  |  |
| *G. b. g.* | 0.368 | - |  | *G. b. g.* | 0.115 | - |  | *G. b. g.* | nd | - |  |
| *G. g. g.* | 0.961 | 0.224 | - | *G. g. g.* | 0.242 | 0.268 | - | *G. g. g.* | nd | 0.203 | *- |

*nd = not determined

**Table S4.** Pairwise effect size among taxa per each wear classes based on the univariate distributions of buccal phase I, lingual phase I and phase II facet relative areas.

|  | **Wear 1** | |  |  | **Wear 2-3** | |  |  | **Wear 4** | |  |
| --- | --- | --- | --- | --- | --- | --- | --- | --- | --- | --- | --- |
| **Buccal** |  | |  | **Buccal** |  | |  | **Buccal** |  | |  |
|  | **G.b.g.** | | **G.b.b.** |  | **G.b.g.** | | **G.b.b.** |  | **G.b.g.** | | **G.b.b.** |
| G.b.b. | -0.36 | |  | G.b.b. | -0.51 | |  | G.b.b. | NA | |  |
| G.g.g. | -0.43 | | 0.08 | G.g.g. | -0.09 | | 0.34 | G.g.g. | 0.33 | | NA |
| **Lingual** |  | |  | **Lingual** |  | |  | **Lingual** |  | |  |
|  | **G.b.g.** | | **G.b.b.** |  | **G.b.g.** | | **G.b.b.** |  | **G.b.g.** | | **G.b.b.** |
| G.b.b. | **0.97** | |  | G.b.b. | **-1.26** | |  | G.b.b. | NA | |  |
| G.g.g. | 0.8 | | -0.07 | G.g.g. | -0.23 | | 0.78 | G.g.g. | **-1.18** | | NA |
| **Phase II** | |  |  | **Phas eII** | |  |  | **Phase II** | |  |  |
|  | | **G.b.g.** | **G.b.b.** |  | | **G.b.g.** | **G.b.b.** |  | | **G.b.g.** | **G.b.b.** |
| G.b.b. | -0.7 | |  | G.b.b. | **1.07** | |  | G.b.b. | NA | |  |
| G.g.g. | -0.8 | | -0.18 | G.g.g. | 0.39 | | **-0.93** | G.g.g. | 0.7 | | NA |

**Table S5.** Tukey Honest Significant Differences test on the analysis of multivariate homogeneity of group dispersion in facet inclination for each wear class. Lower and upper values refer to 95% confidence intervals.

|  | **difference** | **lower** | **upper** | **adj. p-value** |
| --- | --- | --- | --- | --- |
| **2_1** | -1.92 | -5.55 | 1.7 | 0.51 |
| **3_1** | -1.82 | -5.45 | 1.8 | 0.56 |
| **4_1** | -0.53 | -5.03 | 3.9 | 0.99 |
| **3_2** | 0.1 | -2.96 | 3.2 | 1 |
| **4_2** | 1.4 | -2.67 | 5.5 | 0.8 |
| **4_3** | 1.3 | -2.77 | 5.4 | 0.84 |

**Table S6.** Tukey Honest Significant Differences test on the analysis of multivariate homogeneity of group dispersion of wear facet inclinations between mountain gorillas (*G. b. b.*), Grauer’s gorillas (*G. b. g.*) and western lowland gorillas (*G. g. g*.). Lower and upper values refer to 95% confidence intervals.

| **Groups** | **difference** | **lower** | **upper** | **adj. p-value** |
| --- | --- | --- | --- | --- |
| G.b.b. - G.b.g. | 2.39 | -2.65 | 7.43 | 0.5 |
| G.g.g. - G.b.g. | 0.75 | -2.34 | 3.85 | 0.83 |
| G.g.g. - G.b.b. | -1.64 | -6.16 | 2.89 | 0.67 |

**Table S7.** Between-group comparisons (Mann-Whitney test) of wear facet inclinations. Mountain gorillas (*G. b. b.*), eastern lowland gorillas (*G. b. g.*) and western lowland gorillas (*G. g. g*.). Significant differences at *p*<0.05 are highlighted in bold.

| **Wear 1** |  |  |  | **Wear 2** |  |  |  | **Wear 3** |  |  |  | **Wear 4** |  |  |  |
| --- | --- | --- | --- | --- | --- | --- | --- | --- | --- | --- | --- | --- | --- | --- | --- |
| **Buccal** | **G. b. b.** | **G. b. g.** | **G. g. g.** | **Buccal** | **G. b. b.** | **G. b. g.** | **G. g. g.** | **Buccal** | **G. b. b.** | **G. b. g.** | **G. g. g.** | **Buccal** | **G. b. b.** | **G. b. g.** | **G. g. g.** |
| G. b. b. | - |  |  | G. b. b. | - |  |  | G. b. b. | - |  |  | G. b. b. | - |  |  |
| G. b. g. | 0.551 | - |  | G. b. g. | nd* | - |  | G. b. g. | nd | - |  | G. b. g. | nd | - |  |
| G. g. g. | 0.200 | 0.687 | - | G. g. g. | nd | **0.016** | - | G. g. g. | nd | 0.798 | - | G. g. g. | nd | 0.944 | - |
| **Lingual** | **G. b. b.** | **G. b. g.** | **G. g. g.** | **Lingual** | **G. b. b.** | **G. b. g.** | **G. g. g.** | **Lingual** | **G. b. b.** | **G. b. g.** | **G. g. g.** | **Lingual** | **G. b. b.** | **G. b. g.** | **G. g. g.** |
| G. b. b. | - |  |  | G. b. b. | - |  |  | G. b. b. | - |  |  | G. b. b. | - |  |  |
| G. b. g. | 0.233 | - |  | G. b. g. | nd | - |  | G. b. g. | nd | - |  | G. b. g. | nd | - |  |
| G. g. g. | 0.693 | 0.139 | - | G. g. g. | nd | 0.884 | - | G. g. g. | nd | 0.584 | - | G. g. g. | nd | 0.525 | - |
| **Phase II** | **G. b. b.** | **G. b. g.** | **G. g. g.** | **Phase II** | **G. b. b.** | **G. b. g.** | **G. g. g.** | **Phase II** | **G. b. b.** | **G. b. g.** | **G. g. g.** | **Phase II** | **G. b. b.** | **G. b. g.** | **G. g. g.** |
| G. b. b. | - |  |  | G. b. b. | - |  |  | G. b. b. | - |  |  | G. b. b. | - |  |  |
| G. b. g. | 0.371 | - |  | G. b. g. | nd | - |  | G. b. g. | nd | - |  | G. b. g. | nd | - |  |
| G. g. g. | 0.844 | 0.420 | - | G. g. g. | nd | 0.688 | - | G. g. g. | nd | 0.289 | - | G. g. g. | nd | 0.832 | - |
| **Tip crush** | **G. b. b.** | **G. b. g.** | **G. g. g.** | **Tip crush** | **G. b. b.** | **G. b. g.** | **G. g. g.** | **Tip crush** | **G. b. b.** | **G. b. g.** | **G. g. g.** | **Tip crush** | **G. b. b.** | **G. b. g.** | **G. g. g.** |
| G. b. b. | - |  |  | G. b. b. | - |  |  | G. b. b. | - |  |  | G. b. b. | - |  |  |
| G. b. g. | 0.428 | - |  | G. b. g. | nd | - |  | G. b. g. | nd | - |  | G. b. g. | nd | - |  |
| G. g. g. | 0.299 | 1 | - | G. g. g. | nd | 0.182 | - | G. g. g. | nd | 0.401 | - | G. g. g. | nd | 0.832 | - |

*nd = not determined

**Table S8.** Permanova on the impact of the interaction between wear class and taxonomy on wear facet inclinations across mountain gorillas (*G. b. b.*), Grauer’s gorillas (*G. b. g.*), and western lowland gorillas (*G. g. g*.).

|  | **Df** | **SS** | **R2** | **F** | **p-value** |
| --- | --- | --- | --- | --- | --- |
| Wear | 3 | 3749.7 | 0.18874 | 8.3874 | 0.001 |
| Taxonomy | 2 | 258.7 | 0.01302 | 0.868 | 0.527 |
| Wear:Taxonomy | 6 | 956.8 | 0.04816 | 1.07 | 0.365 |
| Residual | 100 | 14902.3 | 0.75008 |  |  |
| Total | 111 | 19867.5 | 1 |  |  |

**Table S9.** Kruskal-Wallis test of differences in occlusal relief index (OR), percentage of dentine exposure (PDE), and percentage of enamel wear (PEW) between mountain gorillas (*G. b. b.*), Grauer’s gorillas (*G. b. g.*), and western lowland gorillas (*G. g. g*.). Significant results are highlighted in bold.

| **Variable** | **KW Χ^2^** | **df** | **p-value** |
| --- | --- | --- | --- |
| OR | 0.11 | 2 | 0.94 |
| PDE | 3.26 | 2 | 0.19 |
| PEW | 8.2 | 2 | **0.016** |

**Table S10.** Dunn test with Benjamini-Hochberg corrected p-values on pairwise differences in the percentage of enamel wear (PEW) between mountain gorillas (*G. b. b.*), Grauer’s gorillas (*G. b. g.*), and western lowland gorillas (*G. g. g*.).

| **Groups** | **Z** | **adj. p-value** |
| --- | --- | --- |
| G.b.b. - G.b.g. | 0.8196745 | 0.4124017 |
| G.g.g. - G.b.g. | -2.0487435 | 0.0607308 |
| G.g.g. - G.b.b. | -2.3154136 | 0.06177092 |

**Table S11.** Between-group comparisons (two-tailed Mann-Whitney tests for independent study design) of relative wear areas of male and female western lowland gorillas.

|  | **Buccal** | | **Lingual** | | **Phase II** | |
| --- | --- | --- | --- | --- | --- | --- |
| **Wear** | **W** | **p-value** | **W** | **p-value** | **W** | **p-value** |
| Wear 1 | 11.5 | 0.9 | 3.5 | 0.14 | 12 | 0.82 |
| Wear 2+3 | 261.5 | 0.72 | 262.5 | 0.73 | 283 | 0.94 |

**Table S12.** Tukey Honest Significant Differences test on the analysis of multivariate homogeneity of group dispersion of wear facet inclinations (Buccal PI, Lingual PI, Phase II and Tip crush) in male and female western lowland gorillas. Lower and upper values refer to 95% confidence intervals.

| **Wear** | **difference** | **lower** | **upper** | **adj. p-value** |
| --- | --- | --- | --- | --- |
| Wear 1 | -0.74 | -13.76 | 12.28 | 0.9 |
| Wear 2 | -1.99 | -9.82 | 5.83 | 0.6 |
| Wear 3 | -1.81 | -5.69 | 2.06 | 0.34 |

**Table S13.** Permanova of wear facet inclinations (Buccal PI, Lingual PI, Phase II and Tip crush) in male and female western lowland gorillas for each wear class.

| **Wear** | **F** | **df** | **P-value** |
| --- | --- | --- | --- |
| Wear 1 | 1.1738 | 1 | 0.32 |
| Wear 2 | 2.35 | 1 | 0.09 |
| Wear 3 | 0.79 | 1 | 0.51 |

**Table S14.** Two-tailed Mann-Whitney test for independent study design on differences in occlusal relief index (OR), percentage of dentine exposure (PDE), and percentage of enamel wear (PEW) between male and female western lowland gorillas.

| **Variable** | **W** | **p-value** |
| --- | --- | --- |
| OR | 364 | 0.58 |
| PDE | 388 | 0.33 |
| PEW | 440.5 | 0.055 |

**Table S15.** List of teeth according to wear stage from gorilla specimens included in this study with information about taxonomy, age, sex and locality from museum records (Cooper and Gordon, 2017).

| **Specimen** | **Taxon** | **Tooth** | **Wear^a^** | **Age** | **Sex** | **Locality** | **Repository**^b^ |
| --- | --- | --- | --- | --- | --- | --- | --- |
| BM 22.2.10.2 | *G. b. beringei* | LM2 | 1 | Adult | F | British Rwanda, Kigezy District | NHML |
| RG 2258 | *G. b. beringei* | RM2 | 1 | Adult | F | R.D. Congo, Virunga Mt. | RMCA |
| 621445 | *G. b. beringei* | RM2 | 1 | Adult | M | Mount Karisimbi, Virunga | NHMS |
| 631164 | *G. b. beringei* | LM2 | 1 | Adult | M | Mount Mikeno, Virunga | NHMS |
| RG 8608 | *G. b. beringei* | LM2 | 1 | Adult | F | R.D. Congo, Karisimbi Mt. | RMCA |
| RG 8607 | *G. b. beringei* | RM2 | 2 | Adult | F | R.D. Congo, Karisimbi Mt. | RMCA |
| 641165 | *G. b. beringei* | LM2 | 2 | Subadult | M | Mount Mikeno, Virunga | NHMS |
| SMF 94796 | *G. b. beringei* | LM2 | 3 | Adult | M | Rwanda | SRI |
| 630739 | *G. b. beringei* | RM2 | 3 | Adult | F | Mount Mikeno, Virunga | NHMS |
| RG2263 | *G. b. beringei* | LM2 | 4 | Adult | F | R.D. Congo, Virunga Mt. | RMCA |
| RG 833 | *G. b. graueri* | RM2 | 1 | Adult | F | R.D. Congo, Baraka | RMCA |
| RG 1002 | *G. b. graueri* | RM2 | 1 | Subadult | F | R.D. Congo, Baraka | RMCA |
| RG 26206 | *G. b. graueri* | LM2 | 2 | Adult | M | R.D. Congo, Bukavu | RMCA |
| RG 881 | *G. b. graueri* | RM2 | 1 | Subadult | F | R.D. Congo, Baraka | RMCA |
| RG 14615 | *G. b. graueri* | RM2 | 2 | Adult | F | R.D. Congo, Lubero | RMCA |
| RG 22761 | *G. b. graueri* | LM2 | 2 | Adult | M | R.D. Congo, Chibimbi Katana | RMCA |
| RG 998 | *G. b. graueri* | LM2 | 2 | Adult | M | Baraka, R.D. Congo | RMCA |
| ZMB 31625 | *G. b. graueri* | LM2 | 2 | Adult | F | 80km NW v. Boko | ZMB |
| 115609 | *G. b. graueri* | LM2 | 2 | Adult | M | Alimbongo Mountains, Congo | AMNH |
| RG 23436 | *G. b. graueri* | LM2 | 2 | Adult | F | Tshela region | RMCA |
| RG 9405 | *G. b. graueri* | RM2 | 2 | Adult | M | Katana, R.D.Congo | RMCA |
| RG 15356 | *G. b. graueri* | RM2 | 2 | Adult | F | Ibatsero, R.D. Congo | RMCA |
| 86044 M7 | *G. b. graueri* | LM2 | 3 | Adult | F | Mungele, R.D Congo | RMCA |
| RG 24001 | *G. b. graueri* | RM2 | 3 | Adult | M | R.D. Congo, Kiwa Lowa | RMCA |
| RG 17770 | *G. b. graueri* | LM2 | 3 | Adult | M | R.D. Congo, Lubutu | RMCA |
| RG 999 | *G. b. graueri* | LM2 | 3 | Adult | M | Baraka, R.D. Congo | RMCA |
| RG 14769 | *G. b. graueri* | LM2 | 3 | Adult | F | Shabunda, R.D. Congo | RMCA |
| 86044 M5 | *G. b. graueri* | RM2 | 3 | Adult | M | Mungele, R.D Congo | RMCA |
| 86044 M8 | *G. b. graueri* | RM2 | 4 | Adult | F | R.D. Congo, Mungele | RMCA |
| 86044 M17 | *G. b. graueri* | LM2 | 4 | Adult | M | R.D. Congo, Kasese | RMCA |
| RG 14770 | *G. b. graueri* | RM2 | 3 | Adult | F | R.D. Congo, Shabunda | RMCA |
| RG 15234 | *G. b. graueri* | LM2 | 3 | Adult | M | R.D. Congo, Warega | RMCA |
| RG 15352 | *G. b. graueri* | RM2 | 4 | Adult | F | R.D. Congo, Ibatsero | RMCA |
| RG 11725 | *G. b. graueri* | LM2 | 4 | Adult | F | Lubero, R.D. Congo | RMCA |
| BM 86.534 | *G. g. gorilla* | LM2 | 1 | Adult | M | Congo | NHML |
| 7732 M16 | *G. g. gorilla* | LM2 | 1 | Subadult |  | Cameroon, Moboe | RMCA |
| 7221 | *G. g. gorilla* | LM2 | 1 | Adult | F | Gabon | PRI |
| ZMB 7157 | *G. g. gorilla* | LM2 | 1 | Subadult | F | Samakita, Ogowe, Gabon | ZMB |
| ZMB 10493 | *G. g. gorilla* | RM2 | 1 | Adult | M | Mayombe, French Congo | ZMB |
| ZMB 30893 | *G. g. gorilla* | LM2 | 1 | Subadult | M | Ajoshone, Akonolinga, Cameroon | ZMB |
| ZMB 31435 | *G. g. gorilla* | LM2 | 1 | Adult | F | Ajoshone, Cameroon | ZMB |
| ZMB 47526 | *G. g. gorilla* | RM2 | 1 | Adult | M | Molundu, Cameroon | ZMB |
| ZMB 83521 | *G. g. gorilla* | RM2 | 1 | Subadult | M | Molundu, Cameroon | ZMB |
| BM 86.537 | *G. g. gorilla* | RM2 | 1 | Subadult | M | Congo | NHML |
| 167333 | *G. g. gorilla* | LM2 | 1 | Adult | M | Cameroon | AMNH |
| 7556 M15 | *G. g. gorilla* | RM2 | 1 | Adult |  | Cameroon, Djaposten | RMCA |
| ZMB 31702 | *G. g. gorilla* | RM2 | 1 | Subadult | F | Bipindi, Cameroon | ZMB |
| SMF 21H248 | *G. g. gorilla* | LM2 | 2 | Adult |  | Unknown | SRI |
| SMF 1135 | *G. g. gorilla* | LM2 | 2 | Subadult | M | Gabon, Ogooue-Maritime | SRI |
| SMF 45713 | *G. g. gorilla* | RM2 | 2 | Adult |  | Cameroon | SRI |
| BM 36.7.14.1 | *G. g. gorilla* | LM2 | 2 | Adult | M | British Cameroons | NHML |
| BM 48.435 | *G. g. gorilla* | LM2 | 2 | Adult | M | Cameroon, Mamfe | NHML |
| 7732 M2 | *G. g. gorilla* | LM2 | 2 | Adult | M | Cameroon, Mimbomimbo | RMCA |
| 7732 M7 | *G. g. gorilla* | LM2 | 2 | Adult | M | Cameroon, Mimbomimbo | RMCA |
| 7732 M8 | *G. g. gorilla* | LM2 | 2 | Adult | M | Cameroon, Mimbomimbo | RMCA |
| 7732 M18 | *G. g. gorilla* | RM2 | 2 | Adult | M | Cameroon, Moboe | RMCA |
| RG 63 | *G. g. gorilla* | RM2 | 2 | Adult |  | Cameroon, Djaposten | RMCA |
| SMF 4108 | *G. g. gorilla* | LM2 | 2 | Adult | M | Cameroon | SRI |
| 90194 | *G. g. gorilla* | RM2 | 2 | Adult | M | Cameroon, East, Moloundou N'Guilili | AMNH |
| 214113 | *G. g. gorilla* | RM2 | 2 | Adult |  | R.D. Congo, Brazaville | AMNH |
| ZMB 6964 | *G. g. gorilla* | RM2 | 2 | Subadult |  | Gabon | ZMB |
| ZMB 11642 | *G. g. gorilla* | LM2 | 2 | Adult | M | Yaunde, Cameroon | ZMB |
| ZMB 14644 | *G. g. gorilla* | RM2 | 2 | Adult | F | Loango, French Congo | ZMB |
| ZMB 20318 | *G. g. gorilla* | RM2 | 2 | Adult |  | Umgeb. U. Nginda, Molundu, Cameroon | ZMB |
| ZMB 30892 | *G. g. gorilla* | RM2 | 2 | Adult | F | Ajoshone, Cameroon | ZMB |
| 167334 | *G. g. gorilla* | RM2 | 2 | Adult | M | Cameroon | AMNH |
| 167337 | *G. g. gorilla* | LM2 | 2 | Adult | F | Cameroon | AMNH |
| 167338 | *G. g. gorilla* | LM2 | 2 | Adult | M | Cameroon | AMNH |
| 167339 | *G. g. gorilla* | RM2 | 2 | Adult | F | Cameroon | AMNH |
| ZMB 83546 | *G. g. gorilla* | LM2 | 2 | Adult | M | ZW Lomie U. Malen, Cameroon | ZMB |
| ZMB 83561 | *G. g. gorilla* | RM2 | 2 | Adult | M | Lolodorf, Cameroon | ZMB |
| ZMB 11646 | *G. g. gorilla* | RM2 | 2 | Adult |  | Bipindi, Cameroon | ZMB |
| ZMB 30940 | *G. g. gorilla* | RM2 | 2 | Adult | M | Camerun | ZMB |
| 7556 M12 | *G. g. gorilla* | LM2 | 2 | Adult | M | Cameroon, Essengbot | RMCA |
| SMF 1133 | *G. g. gorilla* | LM2 | 3 | Adult | M | Africa | SRI |
| BM 86.535 | *G. g. gorilla* | RM2 | 3 | Adult | M | Congo | NHML |
| BM 1939.928 | *G. g. gorilla* | LM2 | 3 | Adult | M | Gabon | NHML |
| 7732 M3 | *G. g. gorilla* | LM2 | 3 | Adult | F | Cameroon, Masins | RMCA |
| BM 1939.936 | *G. g. gorilla* | LM2 | 3 | Adult | F | Gabon | NHML |
| 7732 M4 | *G. g. gorilla* | RM2 | 3 | Adult |  | Cameroon, Moboe | RMCA |
| 7732 M5 | *G. g. gorilla* | RM2 | 3 | Adult | M | Cameroon, Moboe | RMCA |
| 7732 M13 | *G. g. gorilla* | LM2 | 3 | Adult | F | Cameroon, Dja | RMCA |
| BM 1939.927 | *G. g. gorilla* | LM2 | 3 | Adult | F | Gabon | NHML |
| BM 64.12.1.5 | *G. g. gorilla* | RM2 | 3 | Adult | F | Gabon | NHML |
| 167326 | *G. g. gorilla* | LM2 | 3 | Adult | M | Cameroon | AMNH |
| 167327 | *G. g. gorilla* | RM2 | 3 | Adult | M | Cameroon | AMNH |
| 183131 | *G. g. gorilla* | LM2 | 3 | Adult |  | Cameroon, East, Youkadouma | AMNH |
| 200506 | *G. g. gorilla* | RM2 | 3 | Subadult |  | R.D. Congo, Sangha, Ouesso | AMNH |
| 214104 | *G. g. gorilla* | RM2 | 3 | Adult |  | R.D. Congo, Brazaville | AMNH |
| 214114 | *G. g. gorilla* | RM2 | 3 | Adult |  | R.D. Congo, Brazaville | AMNH |
| 214115 | *G. g. gorilla* | LM2 | 3 | Adult |  | R.D. Congo, Brazaville | AMNH |
| ZMB 11643 | *G. g. gorilla* | RM2 | 3 | Adult | F | Bipindi, Cameroon | ZMB |
| ZMB 14645 | *G. g. gorilla* | RM2 | 3 | Adult | M | Campo 1887, Cameroon | ZMB |
| ZMB 14647 | *G. g. gorilla* | LM2 | 3 | Adult | F | Campo, Cameroon | ZMB |
| ZMB 20306 | *G. g. gorilla* | LM2 | 3 | Adult |  | Umgeb. U. Nginda, Molundu, Cameroon | ZMB |
| ZMB 30943 | *G. g. gorilla* | RM2 | 3 | Adult | F | Unknown | ZMB |
| ZMB 31437 | *G. g. gorilla* | LM2 | 3 | Adult | F | Ajoshone, Cameroon | ZMB |
| ZMB 48172 | *G. g. gorilla* | RM2 | 3 | Adult |  | Lomie, Cameroon | ZMB |
| ZMB 83568 | *G. g. gorilla* | RM2 | 3 | Adult | M | Nginda, Cameroon | ZMB |
| ZMB 83573 | *G. g. gorilla* | RM2 | 3 | Adult |  | Ossidinge, Cameroon | ZMB |
| 167332 | *G. g. gorilla* | LM2 | 4 | Adult | M | Cameroon | AMNH |
| SMF 5277 | *G. g. gorilla* | LM2 | 4 | Adult | F | Cameroon | SRI |
| SMF 59159 | *G. g. gorilla* | LM2 | 4 | Adult |  | Duisburg Zoo | SRI |
| BM 49.603 | *G. g. gorilla* | LM2 | 3 | Adult | M | West Africa | NHML |
| 214107 | *G. g. gorilla* | RM2 | 4 | Adult | M | R.D. Congo, Brazaville | AMNH |
| 7732 M6 | *G. g. gorilla* | RM2 | 4 | Adult | M | Cameroon, Mimbomimbo | RMCA |
| 7732 M20 | *G. g. gorilla* | LM2 | 4 | Adult |  | Cameroon, Moboe | RMCA |
| 90061 M1 | *G. g. gorilla* | LM2 | 4 | Adult |  | Cameroon, Moboe | RMCA |
| ZMB 30938 | *G. g. gorilla* | LM2 | 4 | Adult | M | Bipindi, Camerun | ZMB |
| ZMB 83551 | *G. g. gorilla* | LM2 | 4 | Adult | F | Lobomudung, S. Cameroon | ZMB |
| 167325 | *G. g. gorilla* | RM2 | 4 | Adult | M | Cameroon | AMNH |

^a^Tooth wear stage based on Smith (1984).

^b^AMNH = American Natural History Museum (New York, U.S.A.); NHML = Natural History Museum London (UK); NHMS = Natural History Museum Stockholm (Sweden); PRI = Primate Research Institute (Inuyama, Japan); RMCA = Royal Museum for Central Africa (Tervuren, Belgium); SRI = Senckenberg Research Institute (Frankfurt, Germany); ZMB = Museum für Naturkunde (Berlin, Germany).

**Table S16.** Between-group comparisons (Mann-Whitney test) of relative wear facet areas in western lowland gorillas. Significant differences at *p*<0.05 are highlighted in bold.

| **Buccal PI** | **Wear 1** | **Wear 2** | **Wear 3** | **Wear 4** |
| --- | --- | --- | --- | --- |
| Wear 1 | - |  |  |  |
| Wear 2 | 0.574 | - |  |  |
| Wear 3 | 0.499 | 0.907 | - |  |
| Wear 4 | 0.801 | 0.279 | 0.224 | - |
| **Lingual PI** | **Wear 1** | **Wear 2** | **Wear 3** | **Wear 4** |
| Wear 1 | - |  |  |  |
| Wear 2 | **0.008** | - |  |  |
| Wear 3 | **0.010** | 0.209 | - |  |
| Wear 4 | 0.097 | 0.295 | **0.027** | - |
| **Phase II** | **Wear 1** | **Wear 2** | **Wear 3** | **Wear 4** |
| Wear 1 | - |  |  |  |
| Wear 2 | **0.022** | - |  |  |
| Wear 3 | **0.004** | 0.323 | - |  |
| Wear 4 | 0.102 | 1.000 | 0.205 | - |
